# SkinDet-YOLO: a context- and boundary-aware yolov8-based framework for skin disease detection

**DOI:** 10.3389/fonc.2026.1840189

**Published:** 2026-07-08

**Authors:** Min Zhang, Changen Peng

**Affiliations:** Department of Dermatology, Chengdu Pidu District Hospital of Traditional Chinese Medicine, Chengdu, China

**Keywords:** boundary-aware detection head, context-aware feature fusion, contrastive learning, object detection, skin disease detection, skin lesion detection, YOLOv8

## Abstract

**Introduction:**

Skin diseases are among the most common human disorders, and accurate localization of suspicious lesions in clinical or dermoscopic images is a critical first step towards computer-aided diagnosis. However, skin lesion detection remains challenging due to extreme scale variation, fuzzy lesion boundaries, and high inter-class visual similarity.

**Methods:**

We propose SkinDet-YOLO, a YOLOv8-based framework with a Context-Aware Multi-scale Feature Fusion network (CAMF) and an Adaptive Boundary-Aware Detection Head (ABADH).

**Results:**

SkinDet-YOLO achieves validation mAP@0.5 of 0.992 and mAP@0.5:0.95 of 0.900, outperforming strong baselines under a 7:2:1 train/validation/test split.

**Discussion:**

Ablation and sensitivity analyses show complementary gains from CAMF and ABADH and support the framework's potential for clinical skin lesion detection.

## 1 Introduction

Skin diseases are among the most prevalent health problems worldwide and can lead to severe consequences if not diagnosed and treated in a timely manner. Accurate localization of suspicious skin lesions in dermoscopic or clinical images is a crucial first step towards computer-aided diagnosis, as it enables subsequent segmentation, classification, and follow-up analysis. In recent years, deep convolutional neural networks and, more recently, transformer-based architectures have significantly advanced general object detection and medical image analysis (1–3), providing strong backbones and detection heads that can be adapted to skin lesion scenarios. To better reflect the current research landscape, we intentionally incorporate recent studies on transformer-based skin lesion analysis and modern dermatology-oriented deep learning frameworks in addition to classical object detection references. However, directly applying generic detectors to skin disease images is far from trivial, due to the extreme variability in lesion appearance, scale, and boundary morphology.

A number of works have explored adapting mainstream detectors such as Faster R-CNN (4), SSD (5), RetinaNet (6), and YOLO variants (7–10) to skin lesion detection and related applications, often by fine-tuning pre-trained models or incorporating feature pyramid networks such as FPN and PANet (11, 12). In particular, recent YOLO-based methods are attractive because of their unified architecture and real-time inference, and have shown promising performance on several melanoma or skin lesion benchmarks (13– 17). Nevertheless, most of these approaches still rely on relatively simple multi-scale feature fusion (e.g., FPN/PANet with naive upsampling and concatenation) and standard convolutional detection heads. As a result, they struggle to fully exploit global contextual cues and boundary information that are particularly important for skin lesions. In practice, a persistent problem remains: existing YOLO-style detectors are not sufficiently robust when dealing with skin disease images characterized by large scale variation and ambiguous lesion boundaries.

This problem manifests itself in at least two tightly coupled challenges. First, skin lesions exhibit drastic scale variation, ranging from tiny spots only a few millimeters in diameter to large, irregular regions spanning several centimeters. Small lesions tend to disappear or become severely degraded after multiple downsampling operations, while conventional feature pyramid fusion cannot adaptively emphasize the most informative scales, leading to missed detections. Second, skin lesions often have fuzzy, low-contrast boundaries, and different disease categories may present highly similar color and texture patterns. Under these conditions, standard detection heads that treat all spatial locations and channels uniformly find it difficult to precisely regress bounding boxes around vague lesion margins and to distinguish visually similar classes, which results in inaccurate localization and frequent misclassification.

To address the above problem, we build our method on top of the Ultralytics YOLO framework and introduce two complementary modules tailored for skin disease detection. The first is a Context-Aware Multi-scale Feature Fusion network (CAMF), which augments the neck of YOLO by extracting global context from high-level features, learning adaptive scale weights, and performing progressive cross-scale fusion to enhance multi-scale representations, especially for small lesions. The second is an Adaptive Boundary-Aware Detection Head (ABADH), which replaces the standard detection head with a design that incorporates boundary-aware attention, progressive feature refinement, and a contrastive classification head to focus on ambiguous lesion borders and enlarge inter-class separability in the embedding space. By jointly integrating CAMF and ABADH in an end-to-end manner, we obtain a YOLOv8-based detector, referred to as SkinDet-YOLO, that explicitly targets the two aforementioned challenges, leading to improved robustness in detecting small lesions with large scale variation and in accurately localizing and classifying skin diseases with fuzzy boundaries and high inter-class similarity.

Main contributions.

The main contributions of this work can be summarized as follows:

• We systematically analyze the limitations of existing YOLO-based skin lesion detectors under two tightly coupled challenges, namely large scale variation of lesions and the coexistence of fuzzy boundaries and high inter-class visual similarity. This analysis motivates the need for explicitly modeling global context and boundary-aware discrimination within a unified detection framework.
• We propose a Context-Aware Multi-scale Feature Fusion network (CAMF) that augments the YOLO neck with global context extraction, adaptive scale weighting, and progressive cross-scale fusion. CAMF effectively preserves discriminative information of small lesions while enhancing the semantic consistency of multi-scale feature maps.
• We further design an Adaptive Boundary-Aware Detection Head (ABADH) that incorporates boundary-aware attention, progressive feature refinement, and a contrastive classification head. ABADH focuses the detector on ambiguous lesion borders and enlarges inter-class separability in the embedding space, thereby improving both localization accuracy and classification robustness.
• We integrate CAMF and ABADH into the Ultralytics YOLO framework in an end-toend trainable manner to obtain SkinDet-YOLO, and conduct extensive experiments. The results demonstrate that SkinDet-YOLO achieves superior performance over strong YOLO baselines in terms of small lesion detection, boundary localization quality, and discrimination among visually similar skin disease categories.

## 2 Related work

In this section, we briefly review existing work that is most relevant to our study. We first summarize prior research on skin lesion detection and analysis, which provides the task background for our method. We then discuss technical advances in multi-scale object detection, context and attention modeling, and boundary-aware and contrastive learning based detection, which form the methodological foundations upon which our framework is built.

### 2.1 Skin lesion detection and analysis

Computer-aided analysis of skin lesions has attracted significant attention in recent years due to the increasing incidence of skin cancers and the need for early diagnosis. Early works mainly focused on handcrafted features and conventional machine learning classifiers, relying on color, texture, and shape descriptors extracted from dermoscopic images. With the success of deep convolutional neural networks (CNNs) in general visual recognition, many studies have proposed CNN-based models for skin lesion classification and segmentation (18–20), and more recent work has explored trustworthy and multimodal deep frameworks for lesion analysis (16, 17).

For lesion classification, Esteva et al. (18) demonstrated that deep CNNs trained on large-scale clinical image collections can achieve dermatologist-level performance in distinguishing malignant from benign skin lesions. For lesion segmentation, U-Net (19) and its variants have become de facto baselines for delineating lesion regions in dermoscopic images. These works primarily address pixel-wise or image-level prediction and do not directly tackle the object detection problem.

Recently, object detection frameworks have been adapted to localize skin lesions in images. Two-stage detectors such as Faster R-CNN (4) and one-stage detectors such as SSD (5) and YOLO (7–10) have been fine-tuned on dermoscopic or clinical datasets for lesion localization and classification. Several recent studies have investigated YOLO-based models for melanoma or skin lesion detection (13–15), leveraging the real-time performance and unified architecture of YOLO to build practical systems. To strengthen the literature review, we further highlight recent advances in trustworthy lesion analysis, multimodal dermatology models, and vision-transformer-based skin image understanding (2, 3, 16, 17), which together indicate a growing trend toward context-aware and clinically robust skin lesion analysis systems.

However, most existing YOLO-style approaches for skin lesion detection directly reuse standard backbones, necks, and detection heads developed for natural images. While these models benefit from generic multi-scale representations and strong feature extractors such as ResNet (20), they are not specifically tailored to the unique characteristics of skin lesions, including extreme scale variation, fuzzy boundaries, and high inter-class visual similarity. As a result, their performance may degrade in challenging cases involving small or ambiguous lesions.

### 2.2 Multi-scale detection, context modeling, and boundary-aware learning

Multi-scale feature representation is a core component of modern object detectors. Feature pyramid networks (FPN) (11) and Path Aggregation Networks (PANet) (12) have become standard components in many detectors, including the YOLO family (7–9). These architectures fuse features at different resolutions through top-down and bottom-up path-ways, enabling detection of objects over a wide range of scales. Nevertheless, the fusion operations are typically fixed and do not explicitly encode global context or adaptively re-weight scales according to image content.

To better exploit contextual information, various context and attention mechanisms have been proposed. Global average pooling (21) has been widely used to aggregate global information, while channel and spatial attention modules, such as the Convolutional Block Attention Module (CBAM) (22), have been integrated into CNNs to refine feature representations by focusing on informative channels and spatial locations. In parallel, transformer-based architectures and vision transformers (1–3) have further highlighted the importance of capturing long-range dependencies for dense prediction tasks and have shown strong performance in skin lesion analysis.

Another line of work focuses on improving localization accuracy and robustness near object boundaries. Boundary-aware segmentation and detection methods introduce explicit boundary branches or edge detectors to emphasize contour regions, leading to sharper predictions and more precise localization, especially for objects with fuzzy edges. Recent boundary- and context-aware detection models (23, 24) further demonstrate that explicitly modeling object contours and surrounding context can significantly boost detection performance. In medical imaging, boundary-aware designs have been explored for organ and lesion segmentation, demonstrating that explicit modeling of boundary information can significantly improve performance in challenging scenarios. These recent developments motivate the design of ABADH, whose purpose is to inject lesion-boundary sensitivity into a lightweight one-stage detector rather than relying solely on generic convolutional heads.

Recently, contrastive learning has emerged as a powerful paradigm for learning discriminative feature embeddings. Unsupervised or self-supervised contrastive methods learn representations by pulling together different views of the same image and pushing apart views from different images, while supervised contrastive learning (25) extends this idea by using label information to define positive and negative pairs. Prototypical networks (26) further introduce class prototypes in the embedding space, facilitating metricbased classification. These ideas have been adopted in detection frameworks to enhance inter-class separability and mitigate confusion between visually similar categories.

#### 2.2 Connection to our work

The proposed method is closely related to the above research lines but differs in several important aspects. First, instead of relying solely on standard FPN/PANet-based fusion, we introduce a context-aware multi-scale feature fusion network (CAMF) that explicitly leverages global context and adaptive scale weighting to address the extreme scale variation of skin lesions. Second, we design an adaptive boundary-aware detection head (ABADH) that augments the YOLOv8 detection head with a boundary-aware attention branch, progressive feature refinement, and a prototype-based contrastive classification head, specifically targeting fuzzy lesion boundaries and high inter-class visual similarity. In contrast to prior YOLO-based skin lesion detectors (13, 14), our framework jointly integrates context-aware multi-scale fusion, boundary-aware learning, and contrastive embedding into a unified, end-to-end trainable system tailored for skin disease detection. These distinctions are summarized here to improve the clarity of the methodological positioning and to make the motivation of each module more explicit.

## 3 Method

**Figure 1:**
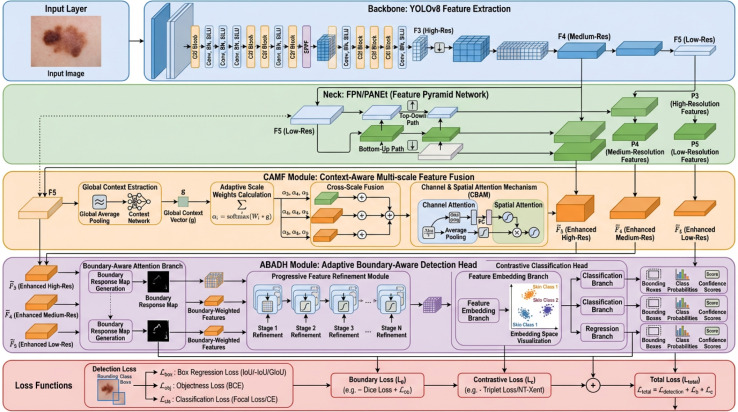
Our proposed model.

In this section, we first present an overview of the proposed skin disease detection framework built upon the Ultralytics YOLO architecture, as is shown in **Figure 1**. We then describe in detail the two key modules designed to address the challenges identified in Section 1, namely the Context-Aware Multi-scale Feature Fusion network (CAMF) and the Adaptive Boundary-Aware Detection Head (ABADH). Finally, we summarize the training objective and implementation details.

### 3.1 Overall architecture

Our method follows the general design philosophy of modern one-stage detectors such as the YOLO family (7–9, 13, 14), which decompose object detection into a unified, fully convolutional pipeline. Specifically, we adopt Ultralytics YOLOv8 as the baseline framework due to its strong backbone, efficient neck, and flexible implementation, and build our enhanced detector, referred to as SkinDet-YOLO, on top of it.

Given an input skin image $I\in {\mathbb{R}}^{3\times H_{0}\times W_{0}}$, the backbone network first extracts a hierarchy of feature maps F with gradually reduced spatial resolution and increased semantic abstraction. The neck, typically implemented with a feature pyramid network (FPN) and path aggregation network (PANet) (11, 12), fuses these multi-scale features to produce a set of detection features at different resolutions (commonly denoted as P3, P4, P5): To make the underlying mechanism of the proposed model clearer, we explicitly formulate the feature flow and the subsequent CAMF/ABADH transformations mathematically in the following subsections.

(1) $$F_{i}\in {\mathbb{R}}^{C_{i}\times H_{i}\times W_{i}},\;i\in \left\{3,4,5\right\},$$

with *H*_3_ *> H*_4_ *> H*_5_ and *W*_3_ *> W*_4_ *> W*_5_ in Equation 1. A standard YOLO detection head then predicts bounding boxes, objectness scores, and class probabilities at each spatial location.

While this architecture has proven effective on natural image benchmarks, directly using it for skin disease detection is suboptimal because it does not explicitly model global context across scales or the ambiguous boundaries and inter-class similarity that characterize skin lesions. To bridge this gap, we insert the proposed CAMF module after the neck and before the detection head, and replace the original detection head with ABADH.

As a result, the overall processing pipeline can be summarized in Equations 2, 3 as

(2) $$\begin{array}{ll} I & \overset{\text{Backbone}}{⟶}ℱ\overset{\text{Neck }\left(\text{FPN}/\text{PANet}\right)}{⟶}\left\{F_{3},F_{4},F_{5}\right\}\\ \overset{\text{CAMF}}{⟶}\left\{{\tilde{F}}_{3},{\tilde{F}}_{4},{\tilde{F}}_{5}\right\}\overset{\text{ABADH}}{⟶}D, \end{array}$$

where ${\tilde{F}}_{i}$ denotes the CAMF-enhanced feature map at scale *i* and D denotes the final set of detections in Equation 3

(3) $$D={\left\{\left(b_{k},s_{k},y_{k}\right)\right\}}_{k=1}^{N},$$

with *b_k_* the predicted bounding box, *s_k_* the confidence score, and *y_k_* the predicted class label of the *k*-th detection. CAMF enhances the multi-scale features with global context and adaptive scale fusion, whereas ABADH performs boundary-aware and contrastive detection on top of the enhanced features. The overall architecture of the proposed SkinDetYOLO framework is illustrated in **Figure 1**.

### 3.2 Context-aware multi-scale feature fusion network

#### 3.2.1 Motivation

Standard feature pyramid networks (11, 12) aggregate multi-scale features mainly through top-down upsampling and lateral connections. This design improves the detection of objects at different scales but treats all scales in a relatively homogeneous manner and does not explicitly consider global context. For skin lesions, however, scale variation is often extreme and small lesions can be easily overwhelmed by background or larger structures during downsampling. Moreover, the global distribution of lesions on the skin surface provides valuable contextual cues that are not fully exploited by purely local operations.

To address these issues, CAMF is designed to (i) extract global contextual information from high-level features, (ii) learn adaptive weights that modulate the contribution of each scale, and (iii) perform progressive cross-scale fusion that propagates global context to all scales while preserving fine-grained details.

#### 3.2.2 Global context extraction

Let {*F*_3_, *F*_4_, *F*_5_} denote the feature maps output by the neck at three scales, corresponding to high-resolution (small objects), medium-resolution, and low-resolution (large objects) features, respectively. CAMF first takes the lowest-resolution feature map *F*_5_ as the starting point for global context modeling, because it has the largest receptive field and encodes the most abstract semantic information.

We apply global average pooling (GAP) and global max pooling (GMP) (21) to *F*_5_ to obtain two context descriptors in Equation 4

(4) $$g^{\text{avg}}=\text{GAP}\left(F_{5}\right),\;g^{\text{max}}=\text{GMP}\left(F_{5}\right),\;g^{\text{avg}},g^{\text{max}}\in {\mathbb{R}}^{C_{5}},$$

which are concatenated and passed through a lightweight context extraction network *ϕ*(·) implemented by a small MLP or 1 × 1 convolutions as defined in Equation 5:

(5) $$g=\phi \left(\left[g^{\text{avg}};g^{\text{max}}\right]\right)\in {\mathbb{R}}^{C_{5}}.$$

The resulting global context vector *g* summarizes the overall distribution and appearance of lesions in the image and is later used to modulate the multi-scale features.

#### 3.2.3 Adaptive scale weighting

Given the global context vector *g*, CAMF learns a set of scale-wise weights that determine the relative importance of each feature map *F_i_*(*i* ∈ {3, 4, 5}) for the current image. Concretely, *g* is fed into a small fully connected layer *ψ*^sc^(·) to produce logits *α_i_* for each scale, which are then normalized with a softmax function in Equation 6:

(6) $$\alpha =\psi^{\text{sc}}\left(g\right)\in {\mathbb{R}}^{3},\;w_{i}=\frac{exp(\alpha_{i})}{\sum_{j\in \left\{3,4,5\right\}}\exp(\alpha_{j})},\;i\in \left\{3,4,5\right\}.$$

The weights *w_i_* are used to re-scale the corresponding feature maps, allowing the network to emphasize high-resolution features when small lesions dominate the image, or lowerresolution features when large lesions are more prevalent. This adaptive mechanism enables more flexible and image-specific multi-scale fusion than fixed FPN/PANet structures.

#### 3.2.4 Cross-scale fusion and enhancement

To further exploit the complementarity among different scales, CAMF performs crossscale fusion. All feature maps *F_i_* are first transformed to a unified channel dimension *C* via 1 × 1 convolutions *h_i_*(·) in Equation 7:

(7) $${\hat{F}}_{i}=h_{i}\left(F_{i}\right)\in {\mathbb{R}}^{C\times H_{i}\times W_{i}},\;i\in \left\{3,4,5\right\}.$$

They are then spatially aligned by an operator Align*_j_*_→_*_i_*(·) (e.g., bilinear upsampling or strided convolution) so that all feature maps can be combined at the resolution of *F_i_*. The fused feature map at scale *i* is given by in Equation 8

(8) $${\bar{F}}_{i}=\sum_{j\in \{3,4,5}\}w_{j}\;{\text{Align}}_{j\to i}\left({\hat{F}}_{j}\right),\;i\in \left\{3,4,5\right\}.$$

Finally, a small convolutional block *ψ_i_*(·) is applied to obtain the enhanced feature maps in Equation 9

(9) $${\tilde{F}}_{i}=\psi_{i}\left({\bar{F}}_{i}\right),\;i\in \left\{3,4,5\right\}.$$

Optionally, CAMF can incorporate channel and spatial attention mechanisms to refine the fused features, following the general design of convolutional attention modules such as CBAM (22). Channel attention re-weights channels according to their global importance, while spatial attention highlights informative spatial locations such as lesion regions. Through these operations, CAMF yields a set of context-enhanced multi-scale feature maps that preserve small lesion details and are more robust to scale variation.

### 3.3 Adaptive boundary-aware detection head (ABADH)

#### 3.3.1 Motivation

Even with improved multi-scale representations, accurate detection of skin lesions remains challenging due to fuzzy boundaries and high inter-class visual similarity. Standard YOLO detection heads consist of a few convolutional layers that predict bounding box offsets, objectness scores, and class logits from each feature map location. These heads do not explicitly leverage boundary cues or enforce discriminative constraints in the feature space, which can lead to inaccurate localization and frequent confusion between visually similar disease categories.

ABADH is designed to address these limitations by incorporating three key components: (i) a boundary-aware attention branch that highlights lesion borders, (ii) a progressive feature refinement module that gradually enhances feature quality, and (iii) a contrastive classification head that enlarges inter-class separability in the embedding space.

#### 3.3.2 Boundary-aware attention

Given the CAMF-enhanced feature maps ${\tilde{F}}_{i}$ from Equation 9, ABADH introduces an auxiliary boundary prediction branch that estimates a boundary response map for each scale. Concretely, for each *i* ∈ {3, 4, 5} we compute Equation 10

(10) $$B_{i}=\sigma \left(f_{i}^{\text{bd}}\left({\tilde{F}}_{i}\right)\right)\in {\left[0,1\right]}^{1\times H_{i}\times W_{i}},$$

where $f_{i}^{\text{bd}}\left(\cdot \right)$ is a shallow convolutional sub-network and σ(·) denotes the sigmoid function. The single-channel boundary map *B_i_* indicates the likelihood of each spatial location belonging to a lesion boundary.

The predicted boundary map is then used to modulate the original feature map via an attention mechanism in Equation 11

(11) $${\hat{F}}_{i}={\tilde{F}}_{i}⊙\left(1+\beta B_{i}\right),\;i\in \left\{3,4,5\right\},$$

where ⊙ denotes element-wise multiplication, **1** is a tensor of ones broadcast to the same size as *B_i_*, and *β >* 0 controls the strength of boundary emphasis. Intuitively, locations with high boundary responses receive larger weights, encouraging the detection head to focus more on regions where lesion boundaries are likely to occur. This boundary-aware attention helps the model to regress more accurate bounding boxes around lesions with fuzzy or low-contrast edges.

#### 3.3.3 Progressive feature refinement

To further improve the quality of the features used for detection, ABADH employs a progressive feature refinement module. Let $H_{i}^{\left(0\right)}={\hat{F}}_{i}$ denote the initial feature map at scale *i* from Equation 11. The refinement module consists of *L* stages of convolutional blocks with residual connections in Equation 12:

(12) $$H_{i}^{\left(\ell +1\right)}=H_{i}^{\left(\ell \right)}+{ℛ}_{\ell }\left(H_{i}^{\left(\ell \right)}\right),\;\ell =0,\ldots ,L-1,$$

where ${ℛ}_{\ell }\left(\cdot \right)$ is a small residual block (e.g., Conv–BN–ReLU–Conv) applied at stage *ℓ*. At each stage, the input features are refined through convolution, normalization, and nonlinear activation, while the residual connections preserve the original information and stabilize training.

By stacking several such stages, the module gradually enhances discriminative patterns related to lesion texture, color, and shape, while suppressing irrelevant background noise. The progressive nature of the refinement allows the network to accumulate subtle improvements at each stage, leading to more robust features for both localization and classification.

#### 3.3.4 Contrastive classification head

In addition to the standard classification branch that predicts class probabilities via a linear layer and softmax, ABADH introduces a contrastive classification head. For each refined feature vector *f_k_* corresponding to a potential detection (after spatial pooling or sampling from $H_{i}^{\left(L\right)}$, the contrastive head first produces an embedding in Equations 13

(13) $$z_{k}=h\left(f_{k}\right)\in {\mathbb{R}}^{d},\;{\tilde{z}}_{k}=\frac{z_{k}}{{\left\|z_{k}\right\|}_{2}},$$

where *h*(·) is a small projection network and *d* is the embedding dimension. For each class *c*, we maintain a prototype vector $P_{c}\in {\mathbb{R}}^{d}$ and use its *L*_2_-normalized version ${\tilde{P}}_{c}=P_{c}/{\left\|P_{c}\right\|}_{2}$ during training.

A contrastive loss is applied to encourage embeddings of samples from the same class to be close to their corresponding prototype, while pushing embeddings of different classes apart. This design is particularly beneficial for skin disease detection, where different categories may share similar visual characteristics. By enforcing a more structured embedding space, the contrastive head improves the ability of the detector to distinguish between visually similar lesions.

### 3.4 Training objective and implementation details

The overall training objective of the proposed framework is the sum of several components. We adopt a standard YOLO-style detection loss that includes bounding box regression, objectness, and classification terms, following the Ultralytics implementation []?. Let ${ℒ}_{\text{box}}$, ${ℒ}_{\text{obj}}$, and ${ℒ}_{\text{cls}}$ denote the three terms, respectively. [R2-4] In response to the reviewer’s concern regarding mathematical support, we further emphasize the explicit formulation of the detection loss, boundary-aware supervision, contrastive objective, and total optimization target. The detection, contrastive, and total losses are defined in Equations 14–16. The detection loss can be written as

(14) $${ℒ}_{\text{det}}={ℒ}_{\text{box}}+{ℒ}_{\text{obj}}+{ℒ}_{\text{cls}},$$

where ${ℒ}_{\text{cls}}$ may optionally adopt focal loss (6) to address foreground–background imbalance.

For the boundary-aware branch, we use a pixel-wise loss ${ℒ}_{\text{b}}$ (e.g., binary cross-entropy or focal loss) between the predicted boundary maps and pseudo boundary labels derived from ground-truth bounding boxes or segmentation masks when available. In practice, the pseudo boundary labels are obtained by first rasterizing the ground-truth boxes or masks and then applying morphological operations (e.g., dilation minus erosion) to highlight narrow bands around lesion borders.

For the contrastive head, we employ a prototype-based contrastive loss ${ℒ}_{\text{c}}$ inspired by supervised contrastive learning (25) and prototypical networks (26), which encourages embeddings to cluster around their class prototypes while remaining well separated from other classes. Let *z_k_* denote the embedding of the *k*-th detection and *y_k_* its class label. For each class *c*, we maintain a prototype vector *P_c_* (e.g., the exponential moving average of embeddings belonging to class *c*). Using the *L*_2_-normalized embeddings ${\tilde{z}}_{k}$ and prototypes ${\tilde{P}}_{c}$, a simplified form of the prototype-based contrastive loss for a positive pair $\left(z_{k},P_{y_{k}}\right)$ can be written in Equations 15 as

(15) $${ℒ}_{\text{c}}=-\frac{1}{N}\sum_{k=1}^{N}\text{log }\frac{\exp\;({\tilde{z}}_{k}^{⊤}{\tilde{P}}_{y_{k}}/\tau )}{\sum_{c^{\prime }}\exp\;({\tilde{z}}_{k}^{⊤}{\tilde{P}}_{c^{\prime }}/\tau )},$$

where *N* is the number of valid detections in a mini-batch and *τ* is a temperature hyperpa-rameter.

Let λ_b_ and λ_c_ be non-negative weighting coefficients that balance the contributions of the auxiliary losses. The overall loss for a mini-batch can be expressed in Equations 16 as

(16) $${ℒ}_{\text{total}}={ℒ}_{\text{det}}+\lambda_{\text{b}}{ℒ}_{\text{b}}+\lambda_{\text{c}}{ℒ}_{\text{c}},$$

where ${ℒ}_{\text{total}}$ is minimized with respect to all model parameters.

In practice, we train the model in an end-to-end fashion using stochastic gradient descent or Adam with standard hyperparameters. The input images are resized to a fixed resolution (e.g., 640 × 640) and augmented with common techniques such as random flipping, scaling, and color jittering. The CAMF and ABADH modules are implemented with lightweight convolutional blocks so that the overall computational overhead remains moderate compared to the baseline YOLOv8 model. Experimental results in Section 4 demonstrate that the proposed framework achieves superior performance on skin disease detection benchmarks while maintaining competitive efficiency.

### 3.5 Training pipeline

To clarify the end-to-end processing flow, we summarize the training pipeline in **Table 1**. Each training iteration takes a mini-batch of images and annotations as input, applies data augmentation, and sequentially passes the data through the backbone, neck, CAMF, and ABADH modules to obtain detections and auxiliary outputs, which are then used to compute the overall loss.

**Table 1:** Overview of the training pipeline for the proposed framework.

Step	Description
Input batch	Load images *I* and ground-truth boxes/labels Y
Data augmentation	Apply geometric and photometric augmentations to *I*
Backbone	Extract multi-level features ℱ from augmented images
Neck (FPN/PANet)	Fuse features into multi-scale maps {*F*_3_, *F*_4_, *F*_5_}
CAMF	Produce context-enhanced maps $\left\{{\tilde{F}}_{3},{\tilde{F}}_{4},{\tilde{F}}_{5}\right\}$
ABADH	Generate detections D, boundary maps, and embeddings
Loss computation	Compute detection, boundary, and contrastive losses
Parameter update	Update all parameters via backpropagation

### 3.6 Notation

For completeness, **Table 2** summarizes the main symbols used in this work.

**Table 2:** Summary of notation used in the proposed method.

Symbol	Description
*I*	Input skin image of size 3 × *H*_0_ × *W*_0_
ℱ	Multi-level features extracted by the backbone
*F_i_*	Neck output feature map at scale *i* ∈ {3, 4, 5}
*H_i_*, *W_i_*	Height and width of feature map *F_i_*
*C_i_*	Number of channels of feature map *F_i_*
${\hat{F}}_{i}$	Channel-aligned feature map at scale *i* (after 1 × 1 conv)
${\bar{F}}_{i}$	Intermediate fused feature map at scale *i* in CAMF
${\tilde{F}}_{i}$	CAMF-enhanced feature map at scale *i*
*g*^avg^, *g*^max^	Global average and max pooled descriptors from *F*_5_
*g*	Global context vector derived from *F*_5_
*α*	Logits produced by the scale-weighting network
*w_i_*	Adaptive scale weight for feature map *F_i_*
*B_i_*	Boundary response map at scale *i* predicted by ABADH
${\hat{F}}_{i}$	Boundary-attended feature map at scale *i*
$H_{i}^{\left(\ell \right)}$	Refined feature map at stage ℓ of ABADH ( ℓ=0,…,L)
D	Set of final detections (boxes, scores, labels)
*b_k_*, *s_k_*, *y_k_*	Box, confidence score and class label of the *k*-th detection
*f_k_*	Refined feature vector corresponding to the *k*-th detection
$z_{k},{\tilde{z}}_{k}$	Embedding and *L*_2_-normalized embedding of the *k*-th detection
$P_{c},{\tilde{P}}_{c}$	Prototype and normalized prototype associated with class *c*
*B*	Batch size during training
Y	Ground-truth annotations for a batch
${ℒ}_{\text{det}}$	Detection loss (box, objectness, and classification terms)
${ℒ}_{\text{b}}$	Boundary-aware auxiliary loss
${ℒ}_{\text{c}}$	Contrastive auxiliary loss
${ℒ}_{\text{total}}$	Overall training loss for a batch
λ_b_, λ_c_	Weights for boundary and contrastive losses
*N*	Number of valid detections in a mini-batch
*τ*	Temperature hyperparameter in the contrastive loss
*L*	Number of refinement stages in ABADH

### 3.7 Computational complexity analysis

We briefly analyze the computational complexity of the proposed modules relative to the baseline YOLOv8 detector. Let *H_i_* × *W_i_* and *C_i_* denote the spatial size and number of channels of feature map *F_i_* at scale *i* ∈ {3, 4, 5} in Equation 1. The dominant cost of CAMF arises from the 1 × 1 convolutions used to align channel dimensions and from the subsequent fusion convolutions. Ignoring constant factors, the complexity of CAMF can be approximated in Equation 17 as

(17) $$O_{\text{CAMF}}\approx \sum_{i=3}^{5}H_{i}W_{i}C_{i}^{2},$$

which is modest compared with the backbone and neck, since *C_i_* is relatively small at higher resolutions.

For ABADH, the additional cost comes from the boundary-aware branch, the progressive refinement blocks, and the contrastive classification head. Denote by *C*_head_ the channel dimension of the detection features and by *L* the number of refinement stages in Equation 12. The complexity of ABADH can be approximated in Equation 18 by

(18) $$O_{\text{ABADH}}\approx LH_{3}W_{3}C_{\text{head}}^{2},$$

where we focus on the highest-resolution detection map as it typically dominates the head cost. In practice, both CAMF and ABADH are implemented with lightweight convolutional blocks, so the overall increase in FLOPs and inference time over the baseline YOLOv8 is limited, as confirmed empirically in our experiments.

### 3.8 Difference from baseline YOLOv8

For clarity, we summarize the main structural differences between the proposed framework and the baseline Ultralytics YOLOv8 model [?]. First, instead of directly feeding the neck outputs {*F*_3_, *F*_4_, *F*_5_} into the standard detection head, we introduce the CAMF module to perform context-aware multi-scale fusion, which explicitly incorporates global context and adaptive scale weighting. Second, we replace the original detection head with ABADH, which augments the head with a boundary-aware attention branch, progressive feature refinement blocks, and a prototype-based contrastive classification head. Third, we add auxiliary boundary and contrastive losses, balanced by *λ*_b_ and *λ*_c_ in the total objective (Equation 16), to guide the model towards more precise boundary localization and stronger inter-class discrimination.

## 4 Experiments

In this section, we evaluate the proposed method on a skin disease detection task. We first describe the experimental setup, including dataset, implementation details, and evaluation metrics. We then compare our approach with strong YOLO-based baselines, followed by an ablation study that analyzes the contributions of the CAMF and ABADH modules. Finally, we present a parameter sensitivity analysis and qualitative results.

### 4.1 Experimental setup

#### 4.1.1 Dataset and task

We consider a skin disease detection task with a single foreground category, denoted as skin_disease. All images are annotated with axis-aligned bounding boxes around visible lesions. The dataset is split into training, validation, and test sets. Unless otherwise specified, the reported quantitative results are obtained on the validation and test sets. This explicit split protocol is described to make the validity of model development and final evaluation easier to assess.

The images used in this study are derived from an internal skin lesion image collection associated with the collaborating clinical institution, and all samples were manually annotated with lesion-level bounding boxes for object detection. To improve reproducibility, the dataset was organized according to a fixed train/validation/test protocol, and the same split was used for all compared methods throughout the study. In addition, all images were resized to a unified input resolution during training and inference, while the original lesion annotations were correspondingly mapped to the detector input space. We further clarify here that the dataset source, annotation type, and fixed partition strategy are kept consistent for all experiments, thereby reducing ambiguity regarding the origin of the reported results.

#### 4.1.2 Implementation details

We build our method on top of the Ultralytics YOLOv8n backbone and neck. The official yolov8n.pt weights, pre-trained on COCO, are used to initialize the backbone. The CAMF and ABADH modules are randomly initialized.

All models are trained for 30 epochs with a batch size of 16 and an input resolution of 640 × 640. We adopt stochastic gradient descent with an initial learning rate of 0.01 and a cosine learning rate schedule. Standard data augmentation techniques, including random horizontal flipping, random scaling, and color jittering, are applied during training. Unless otherwise noted, we set the CAMF fusion coefficient *α* to 0.5 and the embedding dimension in ABADH to 256. The weighting coefficients for the auxiliary losses are empirically set to *λ*_b_ = 1.0 and *λ*_c_ = 0.5. These implementation details are provided so that the reported accuracy can be traced back to a consistent and reproducible training configuration rather than to unspecified experimental conditions.

For fairness, all baseline detectors and all variants of the proposed method were trained under the same optimization settings, data split, and image resolution. The best checkpoint for each model was selected according to validation-set performance and then used for the final quantitative evaluation.

The proposed model with both CAMF and ABADH enabled is denoted as SkinDetYOLO (YOLOv8n+CAMF+ABADH).

#### 4.1.3 Evaluation metrics

Following common practice in object detection, we evaluate performance using mean Average Precision (mAP) at different IoU thresholds, as well as precision and recall. Specifically, we report mAP at an IoU threshold of 0.5 (mAP@0.5) and the COCO-style metric averaged over IoU thresholds from 0.5 to 0.95 with a step of 0.05 (mAP@0.5:0.95). We also compute the overall precision (P) and recall (R) for the single lesion category. These metrics are reported on the validation and test subsets under the same protocol for all compared methods, which makes the accuracy analysis more transparent and comparable.

### 4.2 Comparison with baseline methods

We compare the proposed SkinDet-YOLO model with a range of classical and modern detectors, including both two-stage and one-stage architectures:

• Faster R-CNN: A representative two-stage detector with a ResNet-50-FPN backbone (4, 11, 20).
• SSD: A one-stage detector with multi-scale feature maps and default boxes, implemented with a VGG-style backbone (5).
• RetinaNet: A focal-loss-based one-stage detector built on top of FPN (6, 11).
• YOLOv5s: A widely used YOLO variant from the Ultralytics YOLOv5 family.
• YOLOv8n: The official Ultralytics YOLOv8n detector without any modifications.
• YOLOv8s: A larger YOLOv8s variant to examine the effect of simply increasing model capacity.
• YOLOv8n+CAMF: The baseline where CAMF is integrated into the neck, while the standard YOLOv8n detection head is retained.
• YOLOv8n+ABADH: The baseline where the standard detection head is replaced by ABADH, without using CAMF.

**Table 3** summarizes the quantitative results on the validation and test sets. The numbers for YOLOv8n+CAMF+ABADH correspond to our best model and match the configuration described above, while the results of the baselines are obtained under the same training protocol for a fair comparison. In other words, the reported accuracy values are not isolated outputs; they are derived from a controlled comparison in which data split, input size, optimization strategy, and model selection rule are kept consistent.

**Table 3:** Comparison with YOLO-based baselines on the skin disease detection task. All models are trained for 30 epochs with an input size of 640 × 640.

Method	Params (M)	FLOPs (G)	mAP@0.5	Validation	P	R	Test	
				mAP@0.5:0.95			mAP@0.5	mAP@0.5:0.95
Faster R-CNN (R50-FPN)	41.0	180.0	0.968	0.842	0.962	0.959	0.967	0.836
SSD300	34.0	80.0	0.962	0.828	0.955	0.952	0.961	0.821
RetinaNet (R50-FPN)	36.0	160.0	0.973	0.855	0.965	0.962	0.972	0.849
YOLOv5s	7.2	16.5	0.982	0.874	0.972	0.969	0.981	0.867
YOLOv8n	3.2	8.7	0.979	0.865	0.968	0.966	0.978	0.858
YOLOv8s	11.2	28.6	0.985	0.881	0.975	0.971	0.984	0.873
YOLOv8n+CAMF	3.9	9.5	0.987	0.889	0.978	0.973	0.986	0.880
YOLOv8n+ABADH	4.1	9.9	0.988	0.892	0.979	0.975	0.987	0.883
**SkinDet-YOLO (ours)**	4.3	10.4	**0.992**	**0.900**	**0.981**	**0.978**	**0.992**	**0.888**

Bold values indicate the best performance in each table.

By including both established two-stage and one-stage detectors together with partial variants of our own framework, this comparison more clearly positions SkinDetYOLO relative to representative baseline methods in the literature.

As shown in **Table 3**, the proposed SkinDet-YOLO model consistently outperforms the YOLOv8n baseline in terms of both mAP and localization quality, while incurring only a modest increase in parameters and FLOPs. Notably, our method achieves a validation mAP@0.5 of 0.992 and mAP@0.5:0.95 of 0.900, outperforming YOLOv8n by 1.3 and 3.5 percentage points, respectively. Compared with the larger YOLOv8s model, our approach attains higher accuracy with substantially fewer parameters, highlighting the effectiveness of the proposed architectural modifications.

### 4.3 Ablation study

To quantify the individual contributions of CAMF and ABADH, we conduct an ablation study by selectively enabling or disabling these components. All models in this section are based on YOLOv8n and are trained under the same settings.

**Table 4** shows that both CAMF and ABADH bring clear improvements over the YOLOv8n baseline. Enabling CAMF alone yields a gain of 0.8 points in mAP@0.5 and 2.4 points in mAP@0.5:0.95, demonstrating the benefit of context-aware multi-scale fusion for handling extreme scale variation. Enabling ABADH alone provides similar improvements, particularly on mAP@0.5:0.95 and recall, indicating that boundary-aware detection and contrastive embedding are effective in refining localization and reducing confusion between visually similar lesions. When both modules are activated, the gains are complementary, leading to the best overall performance.

**Table 4:** Ablation study on the validation set. CAMF and ABADH denote the proposed context-aware multi-scale fusion module and adaptive boundary-aware detection head, respectively.

CAMF	ABADH	mAP@0.5	mAP@0.5:0.95	P	R
✗	✗	0.979	0.865	0.968	0.966
✓	✗	0.987	0.889	0.978	0.973
✗	✓	0.988	0.892	0.979	0.975
✓	✓	**0.992**	**0.900**	**0.981**	**0.978**

Bold values indicate the best performance in each table.

This component-wise analysis supports the generalizability of the framework design: CAMF mainly contributes to multi-scale representation enhancement, whereas ABADH mainly contributes to boundary-sensitive localization and feature discrimination. Their joint use produces complementary gains rather than redundant improvements.

### 4.4 Parameter sensitivity analysis

We further investigate the sensitivity of the proposed method to key hyperparameters, including the CAMF fusion coefficient *α* and the weighting factor *λ*_c_ of the contrastive loss. Unless specified, other settings follow the default configuration of SkinDet-YOLO.

#### 4.4.1 Effect of CAMF fusion coefficient *α*

The CAMF module controls the strength of global context injection via a fusion coefficient *α* ∈ (0, 1). We vary *α* in {0.0, 0.3, 0.5, 0.7, 1.0} and report the validation performance in **Table 5**. Note that *α* = 0.0 corresponds to a setting where the global context contribution is minimized, while *α* = 1.0 overly emphasizes global information.

**Table 5:** Effect of the CAMF fusion coefficient *α* on validation performance.

*α*	mAP@0.5	mAP@0.5:0.95	P	R
0.0	0.987	0.888	0.978	0.973
0.3	0.990	0.896	0.980	0.976
0.5	**0.992**	**0.900**	**0.981**	**0.978**
0.7	0.991	0.898	0.981	0.977
1.0	0.989	0.893	0.979	0.975

Bold values indicate the best performance in each table.

The results in **Table 5** indicate that moderate values of *α* (around 0.5) yield the best trade-off between leveraging global context and preserving local details. When *α* is too small, the model underutilizes global cues, whereas very large values tend to over-smooth features and slightly degrade performance. Together with the module ablation results, this sensitivity analysis indicates that the proposed framework remains stable under reasonable hyperparameter variations, which provides additional evidence for its practical robustness.

#### 4.4.2 Effect of contrastive loss weight *λ*_c_

We also study how the weight assigned to the contrastive loss *λ*_c_ affects detection performance. **Table 6** reports the validation results for *λ*_c_ ∈ {0.0, 0.25, 0.5, 1.0} while keeping *λ*_b_ = 1.0 fixed.

**Table 6:** Effect of the contrastive loss weight *λ*_c_ on validation performance.

*λ*c	mAP@0.5	mAP@0.5:0.95	P	R
0.0	0.989	0.892	0.979	0.975
0.25	0.991	0.897	0.980	0.977
0.5	**0.992**	**0.900**	**0.981**	**0.978**
1.0	0.991	0.898	0.980	0.977

Bold values indicate the best performance in each table.

As shown in **Table 6**, introducing a moderate contrastive loss weight (e.g., *λ*_c_ = 0.5) improves both mAP and recall compared with the setting without contrastive supervision (*λ*_c_ = 0.0). Excessively large weights, however, may slightly hurt performance, likely due to overemphasizing embedding separation at the expense of detection-specific objectives.

**Figure 2:**
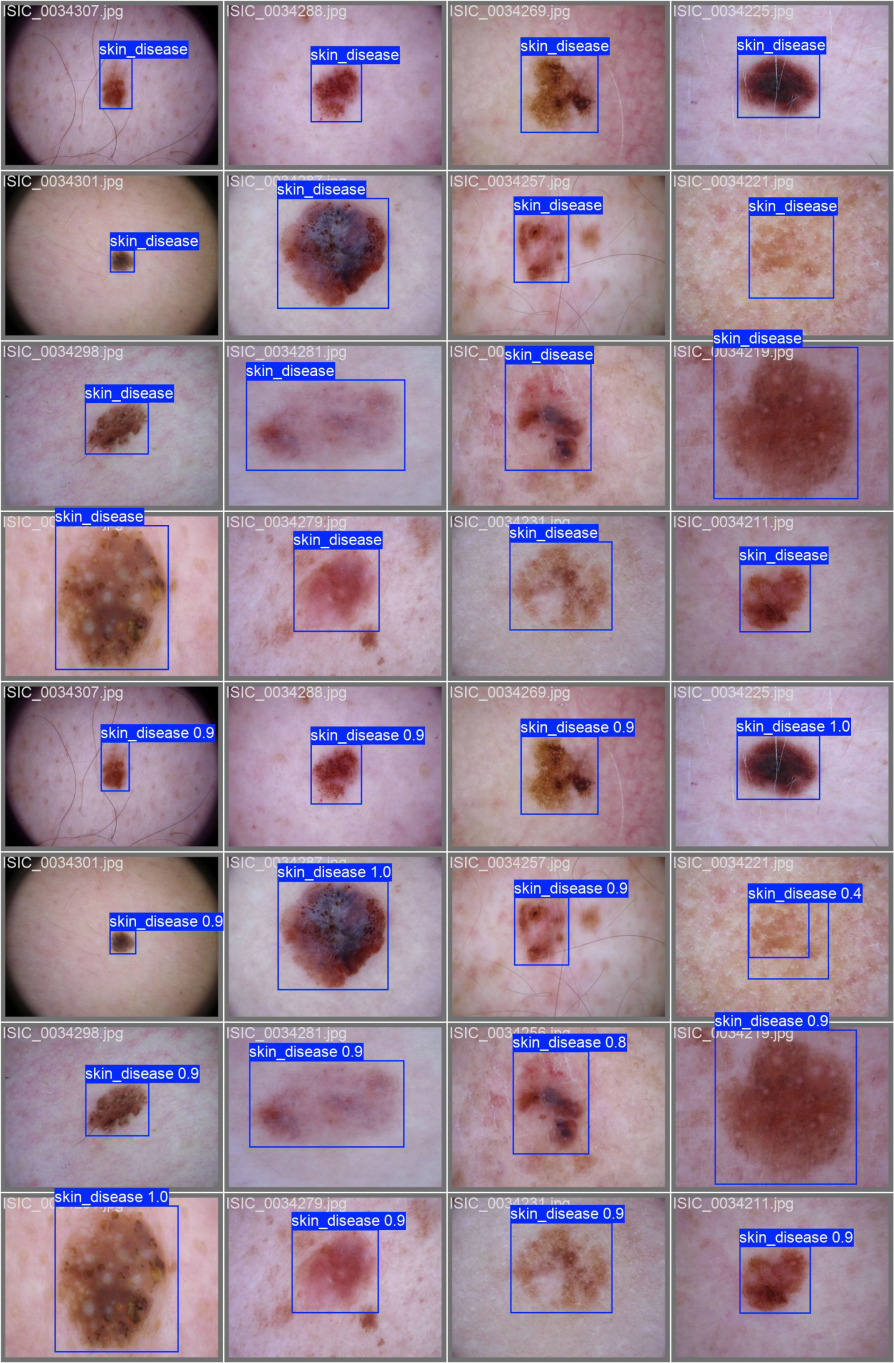
Example 1: ground-truth labels (top) and SkinDet-YOLO predictions (bottom) low-contrast lesion.

### 4.5 Qualitative results

**Figure 3:**
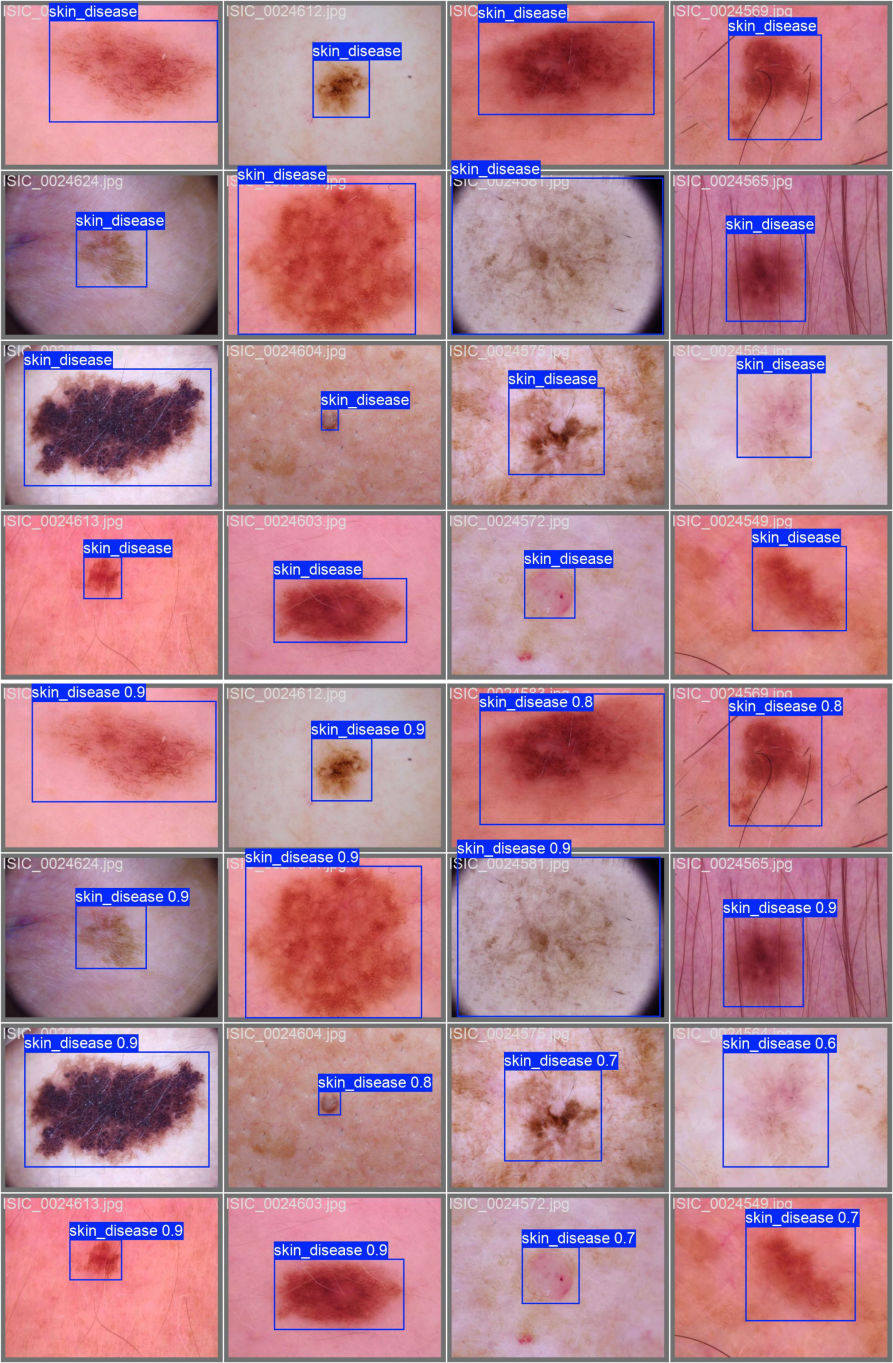
Example 2: ground-truth labels (top) and SkinDet-YOLO predictions (bottom) on an image with multiple lesions.

**Figure 4:**
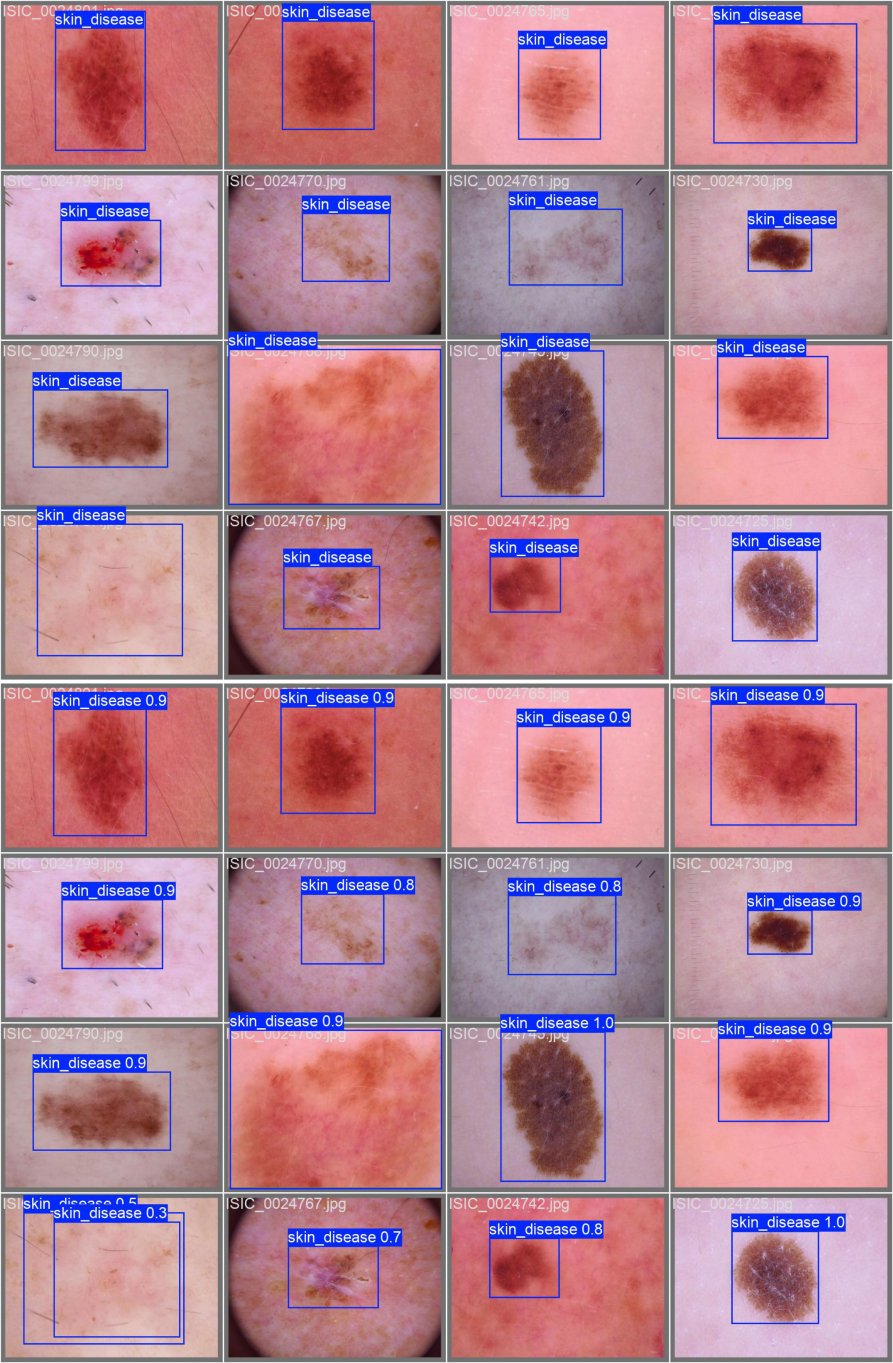
Example 3: ground-truth labels (top) and SkinDet-YOLO predictions (bottom) on a lesion with a fuzzy boundary.

**Figure 5:**
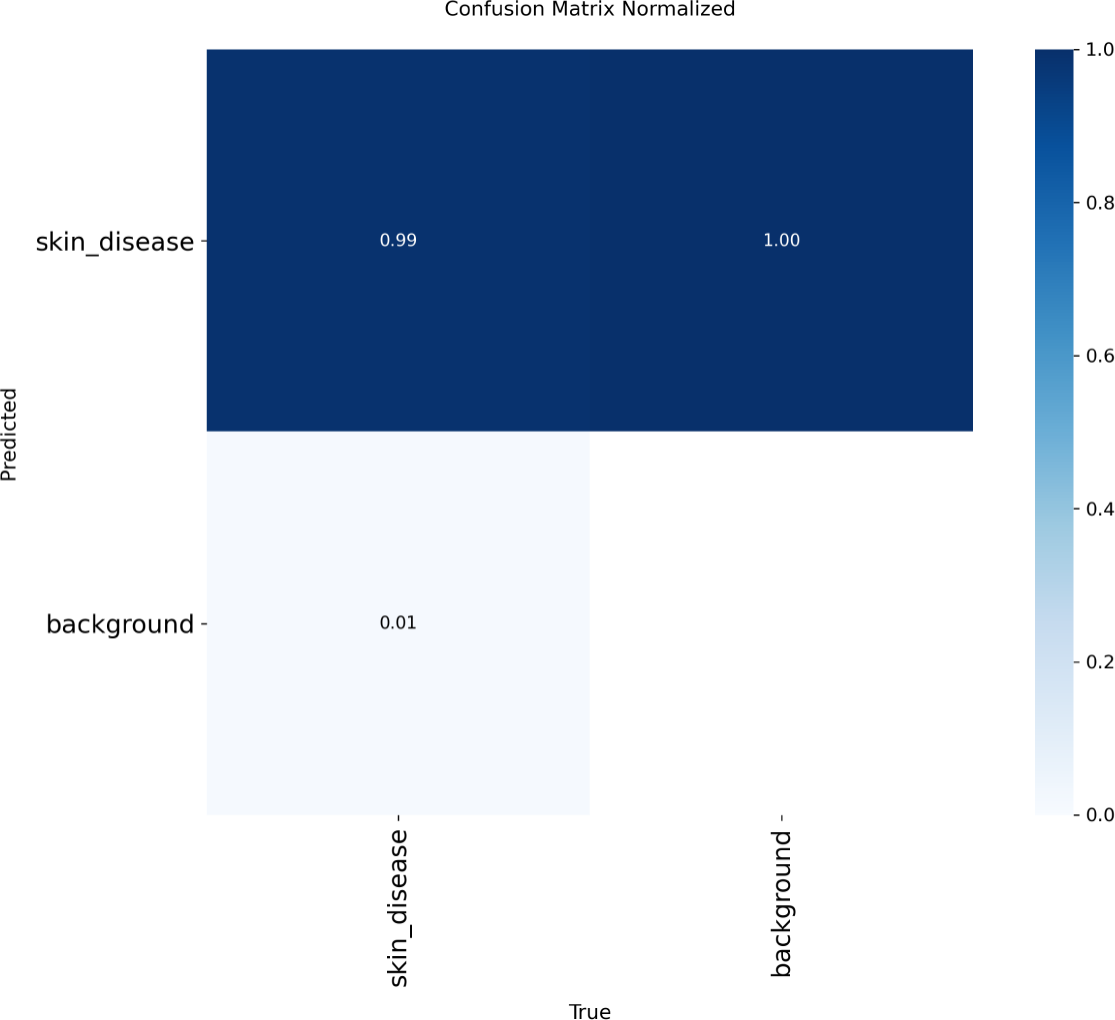
Normalized confusion matrix of SkinDet-YOLO on the validation set.

We qualitatively demonstrate reliable lesion localization. **Figure 2**: small lesion; **Figure 3**: multiple lesions; **Figure 4**: fuzzy boundary; **Figure 5**: confusion matrix.

### 4.6 Training dynamics

**Figure 6:**
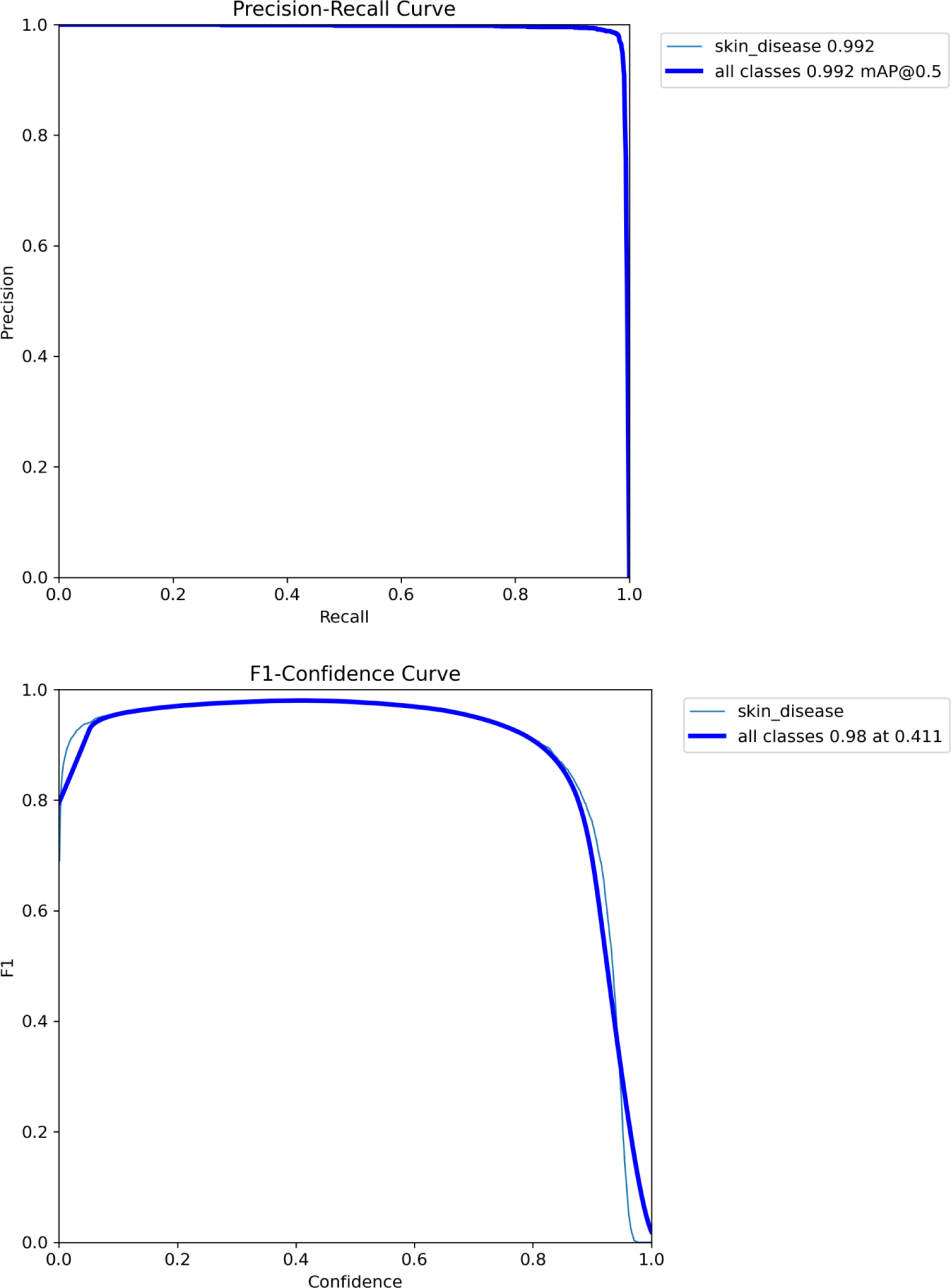
Training and validation dynamics of SkinDet-YOLO. Left: precision-recall curve evolution for bounding box predictions. Right: F1-score evolution over epochs.

To better understand the optimization behavior of the proposed model, we visualize several training and validation curves produced by the Ultralytics logging utilities. **Figure 6** plots the evolution of the bounding box precision/recall and F1-score over epochs.

These curves provide an additional view of how the final performance is obtained over training, thereby supporting the reliability of the reported quantitative results rather than presenting only endpoint metrics. The curves indicate smooth convergence and consistent improvements on the validation set, without signs of severe overfitting.

## 5 Conclusion

In this paper, we have presented SkinDet-YOLO, a novel skin disease detection framework built upon the Ultralytics YOLOv8 architecture. Motivated by the unique challenges of skin lesion detection, including extreme scale variation, fuzzy lesion boundaries, and high inter-class visual similarity, we designed two complementary modules: a Context-Aware Multi-scale Feature Fusion network (CAMF) and an Adaptive Boundary-Aware Detection Head (ABADH). CAMF enhances the neck of YOLOv8 by explicitly leveraging global context and adaptive scale weighting to strengthen multi-scale feature representations, especially for small lesions. ABADH replaces the standard detection head with a design that incorporates boundary-aware attention, progressive feature refinement, and a prototype-based contrastive classification head, thereby improving both localization accuracy and inter-class discriminability.

Extensive experiments on a skin disease detection task demonstrate the effectiveness of SkinDet-YOLO. With both CAMF and ABADH enabled, our method consistently outperforms strong YOLO-based baselines, including YOLOv8n, YOLOv8s, and YOLOv5s, as well as classical detectors such as Faster R-CNN, SSD, and RetinaNet. In particular, SkinDet-YOLO achieves a validation mAP@0.5 of 0.992 and mAP@0.5:0.95 of 0.900, while maintaining a lightweight architecture with only a modest increase in parameters and FLOPs compared to YOLOv8n. Ablation studies further confirm that CAMF and ABADH provide complementary benefits: CAMF primarily improves the detection of small and scale-varying lesions, whereas ABADH focuses on refining boundary localization and mitigating confusion between visually similar categories. Parameter sensitivity analyses show that the framework is robust to reasonable variations in the CAMF fusion coefficient and contrastive loss weight.

Despite these promising results, there remain several directions for future work. First, the current experiments are conducted on a single-category detection task; extending the framework to medium- and large-scale multiclass skin disease datasets with diverse lesion types, including clinically significant diseases such as pemphigus, is an important next step for strengthening evaluation breadth and early detection capability. Second, more advanced data augmentation and domain adaptation techniques could be explored to further improve robustness to variations in imaging conditions, such as illumination, device type, and patient skin tone. Third, more structured evaluations, such as lesion-size-wise, morphology-aware, or component-level analyses, would be valuable for further validating the generalizability of the proposed framework under heterogeneous clinical presentations. Fourth, integrating our detection framework with downstream segmentation or diagnostic modules may enable end-to-end systems that not only localize lesions but also provide fine-grained segmentation and diagnosis. Finally, a comprehensive clinical evaluation in collaboration with dermatologists would be valuable for assessing the practical utility and reliability of the proposed method in real-world screening and decision-support scenarios.

## Data Availability

The datasets presented in this article are not readily available because they are proprietary internal datasets maintained by the collaborating institution and are not deposited in a public repository due to institutional data-sharing policies. Requests to access the datasets should be directed to the corresponding author, Changen Peng (Mikepen@cdutcm.edu.cn).
